# Photoautotrophic Batch Cultivation of *Limnospira* (Spirulina) *platensis*: Optimizing Biomass Productivity and Bioactive Compound Synthesis Through Salinity and pH Modulation

**DOI:** 10.3390/md23070281

**Published:** 2025-07-05

**Authors:** Matteo Rizzoli, Giovanni Antonio Lutzu, Luca Usai, Giacomo Fais, Debora Dessì, Robinson Soto-Ramirez, Bartolomeo Cosenza, Alessandro Concas

**Affiliations:** 1 Department of Life Sciences, University of Modena and Reggio Emilia, Via Giuseppe Campi 287, 41123 Modena, MO, Italy; 287395@studenti.unimore.it; 2 Teregroup Srl, Via David Livingstone 37, 41123 Modena, MO, Italy; gianni.lutzu@teregroup.net (G.A.L.); luca.usai@teregroup.net (L.U.); 3 Department of Mechanical, Chemical and Materials Engineering, University of Cagliari, Piazza d’Armi, 09123 Cagliari, CA, Italy; giacomo.fais@unica.it; 4 Interdepartmental Center of Environmental Science and Engineering (CINSA), University of Cagliari, Via San Giorgio 12, 09124 Cagliari, CA, Italy; 5 Department of Life and Environmental Sciences, University of Cagliari, Cittadella Universitaria, Blocco A, SP8 Km 0.700, 09042 Monserrato, CA, Italy; deboradessi95@gmail.com; 6 Escuela de Ingenieria Bioquimica, Pontificia Universidad Católica de Valparaiso, Avenue Brasil 2085, 72874-220 Valparaíso, Chile; robinson.soto@pucv.cl; 7 Department of Civil and Industrial Engineering, University of Pisa, Largo Lucio Lazzarino, 56122 Pisa, PI, Italy; bartolomeo.cosenza@unipi.it

**Keywords:** *Limnospira platensis*, salinity, alkalinity, phycocianin, polyphenols, antioxidant activity

## Abstract

This study investigates the effects of salinity and pH modulation on the growth, biochemical composition, and bioactive compound production of *Limnospira platensis* under photoautotrophic batch cultivation. Cultures were grown in cylindrical photobioreactors using modified Jourdan medium, with controlled variations in NaCl concentrations (0.2–10 g L^−1^) and pH levels (9–11) to simulate moderate environmental stress. Maximum biomass productivity (1.596 g L^−1^) was achieved at pH 11 with 10 g L^−1^ NaCl, indicating that *L. platensis* can tolerate elevated stress conditions. Phycocyanin (PC) content peaked at 9.54 g 100 g^−1^ dry weight (DW) at pH 10 and 5 g L^−1^ NaCl, triple the value at pH 9, highlighting optimal physiological conditions for pigment synthesis. Protein fraction dominated biomass composition (40–60%), while total lipid content increased significantly under high pH and salinity. Polyphenol content reached 19.5 mg gallic acid equivalents (GAE) gDW^−1^ at pH 10 with 0.2 g L^−1^ NaCl, correlating with the highest antioxidant activity (Trolox equivalent antioxidant capacity). These findings underscore the potential of *L. platensis* as a valuable source of proteins, pigments, and antioxidants, and emphasize the utility of moderate environmental stress in enhancing biomass quality, defined by protein, pigment, and antioxidant enrichment. While this study focused on physiological responses, future research will apply omics approaches to elucidate stress-response mechanisms. This study provides insights into optimizing cultivation strategies for large-scale production exploitable in food, pharmaceutical, and bio-based industries.

## 1. Introduction

Microalgae are emerging as a key focus in biotechnology due to their rapid growth rates, high biomass productivity, and ability to synthesize diverse bioactive compounds such as pigments, polyphenols, lipids, and proteins [1]. These characteristics make them highly versatile for applications including the production of bioethanol, biodiesel, biogas, and biohydrogen, as well as environmental remediation through CO_2_ mitigation strategies, and the development of bioplastics, nutraceuticals, pharmaceuticals, functional foods, and biofertilizers [2,3,4,5]. Moreover, their adaptability to extreme environmental conditions, such as high salinity, high CO_2_ concentrations, extreme temperature, intense UV radiation, acidic or alkaline pH levels, and heavy metal pollution, positions microalgae as an excellent candidate for sustainable and innovative biotechnological solutions [6,7]. Harnessing their potential could significantly advance renewable energy, environmental sustainability, and bio-based product development, fostering innovation across the bio-economy [8]. Among cyanobacteria, *Limnospira platensis* (commonly known as *Spirulina*) is particularly valued for its high protein content, rapid growth, and robustness under diverse environmental conditions [9]. This organism is also recognized for its ability to accumulate pigments, particularly phycocyanin, a blue pigment-protein complex extensively used in the food, cosmetic, and pharmaceutical industries due to its antioxidant, anti-inflammatory, and neuroprotective properties [10,11]. While mixotrophic cultivation has been explored for enhancing phycocyanin production, photoautotrophic cultivation remains a simpler, sustainable, and scalable strategy, particularly in batch systems that enable precise environmental control [12]. Numerous studies have investigated the effects of environmental factors like salinity and pH on the growth and biochemical composition of *L. platensis* [13]. It is well documented that salinity and pH play critical roles in modulating the metabolic and physiological responses of this cyanobacterium. Literature shows how, under stress conditions, microalgae activate specialized metabolic pathways to maintain cellular function. High salinity influences osmoregulation, enzyme activities, and cell membrane integrity [12,14], while pH variations can impact nutrient availability, photosynthetic efficiency, and the biochemical pathways leading to lipid and fatty acid (FA) biosynthesis [15]. Under photoautotrophic conditions, salinity and pH stress significantly impact the lipid profile of *L. platensis*. Elevated salinity induces osmotic stress, leading to the accumulation of compatible solutes and modifications in FA biosynthesis pathways [16]. This stress response often shifts the FA profile toward increased unsaturation, which can enhance the biodiesel quality of the produced lipids [17]. Moreover, changes in pH can influence the enzymatic activity involved in lipid metabolism, thereby altering the overall fatty acid methyl ester (FAME) composition. Recent studies suggest that moderate salinity levels combined with controlled pH can optimize the FA profile for specific industrial applications, including biofuel production and nutraceutical use [18]. For instance, salinity stress has been linked to increased proportions of polyunsaturated fatty acids (PUFAs), which are highly desirable in the health and nutrition sectors. In addition, in high salinity environments, microalgae adjust osmotically by accumulating compatible solutes such as glycine betaine, along with inducing stress-responsive proteins. Conversely, extremely high salinity or pH levels can compromise cell viability and reduce biomass yield, highlighting the importance of precise parameter control [19]. Elevated salinity levels in microalgae induce ionic, osmotic, and oxidative stress [20]. Under salt stress, microalgae generate various reactive oxygen species (ROS), including hydroxyl radicals, hydrogen peroxide, and singlet oxygen. These ROS function as secondary messengers in intracellular signaling pathways, activating adaptive responses to abiotic and biotic stress. Under alkaline conditions, altered CO_2_ availability prompts increased bicarbonate uptake and the activation of photoprotective mechanisms to sustain photosynthesis and energy balance [21].

However, existing research often addresses these factors individually or focuses on extreme salinity levels (>40 g L^−1^), which are less practical for scalable cultivation. Moreover, limited attention has been paid to the combined, moderate adjustment of salinity (0.2–10 g L^−1^ NaCl) and pH (9–11) under strictly photoautotrophic batch conditions, a setting that more closely mimics sustainable large-scale production systems.

Microalgae, including *L. platensis*, contain a diverse range of phenolic compounds that contribute to their antioxidant activity [22]. These bioactive metabolites play a crucial role in physiological processes, particularly in stress adaptation, enabling microalgae to interact with and respond to their environment [23]. These compounds not only exhibit strong antioxidant properties but also contribute to a range of health benefits, including anticancer, antiviral, antimicrobial, anti-inflammatory, and immune-regulating effects [24]. The enhancement of antioxidant production in *Limnospira* under abiotic stress, such as increasing pH levels, has been previously reported [25]. However, the antioxidant response to changes linked to concomitant high alkalinity and pH levels requires further investigation.

This study offers, for the first time, a novel and synergistic investigation into the combined effects of controlled moderate salinity and pH shifts on *L. platensis* cultivated under photoautotrophic batch conditions, a cultivation mode that closely mimics sustainable large-scale production systems but remains underexplored in current literature. Unlike previous research that often focuses on extreme salinity or isolated environmental variables, this work evaluates realistic and scalable stress conditions, capturing their simultaneous influence on biomass productivity and biochemical enrichment. The originality of this study lies in its integrated approach to optimizing multiple high-value traits, namely protein content, pigment synthesis (particularly phycocyanin), lipid accumulation, and antioxidant activity, by fine-tuning environmental stress parameters.

In addition to documenting phenotypic changes, this work provides a mechanistic interpretation of the physiological responses induced by salinity and pH stress, such as osmotic adjustment, activation of antioxidant defenses, and alterations in lipid metabolism. While omics-level analyses were beyond the scope of this study, future research will be directed toward integrating transcriptomics and proteomics to elucidate regulatory networks underlying stress tolerance.

These findings will contribute to broader applications of this cyanobacterium in nutrition and other wealth-related industrial sectors.

## 2. Results

### 2.1. Growth Profile and Biomass Composition of L. platensis in BWW and SW

#### Growth Profile of *L. platensis* Under Salinity and Alkalinity

*L. platensis* was cultivated under photoautotrophic conditions in JM for five days using three different initial alkaline pH levels and three NaCl concentrations. The first concentration, 0.2 g L^−1^ of NaCl, represents a fifth of the salt concentration naturally found in JM, while the other two concentrations, 5 and 10 g L^−1^, are five and ten times higher, respectively. It is important to note that the 0.2 g L^−1^ NaCl condition does not reflect the standard JM composition (which contains 1 g L^−1^ NaCl) but was instead chosen to explore physiological responses under a suboptimal salinity. Therefore, the enhanced growth observed at 5 or 10 g L^−1^ should be interpreted relative to a low-salt stress condition rather than to a full-strength JM baseline. Including a true control at 1 g L^−1^ NaCl is a key improvement planned for subsequent studies. This investigation aimed to evaluate the impact of salinity and pH on key kinetic parameters listed in Table 1, including final biomass concentration ($X_{f}$), volumetric biomass productivity ($Q_{X}$), and average specific growth rate ($\mu_{av}$).

**Figure 1:**
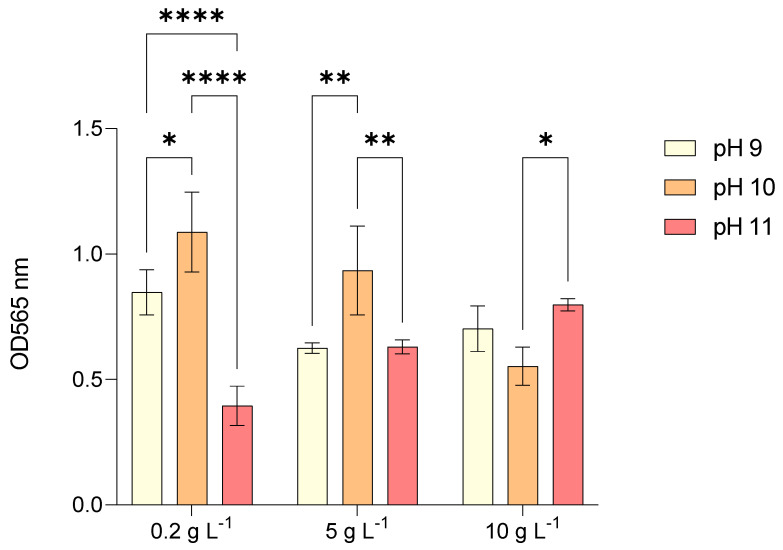
Optical density (OD_565_) of *Limnospira platensis* cultures after 5 days of batch photoautotrophic cultivation under different initial pH (9, 10, 11) and NaCl concentrations (0.2, 5, 10 g L^−1^). Cultures were grown in 20 L cylindrical PBRs (working volume: 3 L), under continuous aeration and a 12 h light/12 h dark photoperiod at room temperature, illuminated with warm white LEDs (150 μmol photons m^−2^ s^−1^). The initial biomass concentration was standardized at 0.3 g L^−1^. Values represent mean ± standard deviation (*n* = 3 biological replicates). Statistical differences were assessed by two-way ANOVA with Tukey’s post hoc test; different asterisks above bars indicate significant differences (* *p* < 0.05, ** *p* < 0.01, *** *p* < 0.001, **** *p* < 0.0001). OD measurements provide a comparative indicator of growth but do not capture lag phase duration or detailed growth kinetics.

Figure 1 depicts the growth of *L. platensis* after five days of cultivation, measured as optical density (OD) at 565 nm under photoautotrophic conditions. The highest OD values, 1.088 and 0.935, were recorded in tests 4 (pH 10 and 0.2 g L^−1^ NaCl) and 5 (pH 10 and 5 g L^−1^ NaCl), respectively. In contrast, at 10 g L^−1^ NaCl, the highest OD (0.798) was observed in test 9 (at pH 11). Overall, when *L. platensis* was cultivated at 0.2 g L^−1^ NaCl, growth at pH 11 (test 7) was more than halved compared to pH 9 (test 1). At 5 g L^−1^ NaCl, growth at pH 10 (test 5) was superior to that at pH 9 (test 2) and 11 (test 8), which exhibited similar growth patterns. Given that the initial OD at day 0 was 0 for all tests, the limited increase in OD observed in test 7 (pH 11 and 0.2 g L^−1^ NaCl) suggests a prolonged lag phase compared to pH 9 and 10. Similarly, in tests 1 (pH 9 and 5 g L^−1^ NaCl) and 8 (pH 11 and 5 g L^−1^ NaCl), the lag phase was estimated to be approximately half the duration observed in test 7. 

In our study, the extended adaptation phase observed in test 7 (and partially in tests 8 and 9) can be attributed to the time required for *L. platensis* to acclimate to the new growth conditions, particularly the higher initial pH compared. Since the natural pH of JM is around 9, an increase of two pH units represents a significant shift. Acclimation is a crucial phase in cyanobacteria adaptation, directly influencing overall culture performance.

*L. platensis* exhibited distinct growth responses to varying salinity and pH levels under photoautotrophic batch cultivation (Figure 1). Initial pH settings (9, 10, and 11) combined with NaCl concentrations (0.2, 5, and 10 g L^−1^) significantly influenced biomass accumulation, though a moderate pH drift toward ~10 was observed across all conditions due to CO_2_ depletion via photosynthesis. This natural pH stabilization should be considered when interpreting the results.

The highest biomass concentration level (1.596 g L^−1^) was achieved with test 9 (pH 11 and 10 g L^−1^ NaCl), whereas the lowest (1.058 g L^−1^) was recorded in test 2 (pH 9 and 5 g L^−1^ of NaCl) (Table 1). When *L. platensis* was grown at the lowest salt concentration under three different pH levels (tests 1, 4, 7), and no significant differences in biomass were observed. However, at both 5 and 10 g L^−1^ NaCl, biomass concentrations varied with increasing pH. Notably, at 10 g L^−1^ NaCl, biomass concentration exhibited a direct proportional relationship with pH (tests 3, 6, and 9), whereas at 5 g L^−1^ NaCl, this trend was only evident between pH 9 and 10 (tests 2 and 5).

Regarding volumetric biomass productivity, the highest $Q_{X}$ value (144 mg L^−1^ d^−1^) was observed in test 6 (pH 10 and 10 g L^−1^ NaCl), while the lowest (82 mg L^−1^ d^−1^) was recorded in test 8 (pH 11 and 5 g L^−1^ NaCl). However, when averaging volumetric biomass productivity across different salinity levels at the same pH, the highest productivity was 0.122 g L^−1^ d^−1^ at pH 9, whereas the lowest was 0.088 g L^−1^ d^−1^ at pH 11. A similar pattern was observed in the difference between the highest and lowest average biomass concentrations throughout the cultivation period, which were 0.72 g L^−1^ and 0.41 g L^−1^, respectively.

For the average specific growth rate ($\mu_{av}$), the highest value (0.172 day^−1^) was recorded in test 2 (pH 9 and 5 g L^−1^ NaCl), which was 1.5 times higher than the lowest $\mu_{av}$ (0.077 day^−1^) observed in test 8 (pH 11 and 5 g L^−1^ NaCl). These findings suggest that higher salinity has an inverse proportional effect on $\mu_{av}$, which tends to increase within the pH 10–11 range.

Although initial pH levels were carefully adjusted at the beginning of each experiment, a moderate drift toward pH ~10 was observed during cultivation across all conditions, primarily due to active photosynthesis and CO_2_ depletion. This phenomenon is typical in photoautotrophic batch systems without continuous pH control. Consequently, while initial pH set points shaped the early phase of growth, final biomass accumulation reflected the combined influence of both the starting and slightly shifted environmental conditions. For this reason, future research will use constant pH control to better understand how starting and stable alkalinity levels affect results. These pH dynamics should also be taken into consideration when interpreting the effects of alkalinity on *L. platensis* physiology.

At low salinity (0.2 g L^−1^ NaCl), growth was highest at pH 9, suggesting that near-neutral alkalinity favored biomass proliferation without imposing significant stress. In contrast, at higher salinities (5 and 10 g L^−1^ NaCl), optimal biomass productivity was recorded at pH 10 (Table 1), indicating that moderate alkaline stress enhanced tolerance to osmotic pressure. However, at pH 11, growth generally declined, especially under 5 g L^−1^ NaCl, suggesting that excessive alkalinity impaired metabolic activity and cell division. These observations can be explained by physiological stress responses; at moderate salinity and alkalinity, *L. platensis* activates osmoprotective mechanisms, such as the synthesis of compatible solutes (e.g., trehalose, glucosylglycerol), stabilization of membrane proteins, and maintenance of photosynthetic efficiency. However, excessive pH (≥11) can lead to impaired carbon assimilation, membrane destabilization, and elevated oxidative stress, ultimately suppressing growth. Salinity stress also likely modulated intracellular ionic balance and triggered antioxidant defenses, as inferred from the enhanced pigment and polyphenol profiles observed at moderate pH 10.

### 2.2. Pigments and Antioxidants Content of L. platensis Under Salinity and Alkalinity

The production of key pigments such as chlorophyll a, carotenoids, and phycocyanin was markedly influenced by the combined effects of pH and salinity. Chlorophyll a content peaked at pH 10 across all tested salinity levels, mirroring the pattern observed for growth. This suggests that pH 10 provided a favorable balance between stress induction and photosynthetic capacity preservation. Elevated alkaline pH levels (10 and 11) enhanced cellular metabolism while maintaining membrane integrity, as evidenced by the kinetic parameters ($Q_{X}$, ΔX and $\mu_{av}$) presented in Table 1, particularly at pH 11.

**Figure 2:**
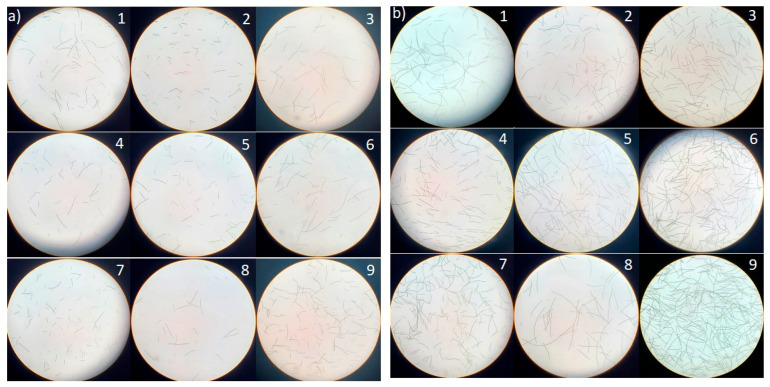
Cytomorphology of *Limnospira platensis* under different salinities and alkalinities taken at 40× magnification at day 0 (**a**) and day 5 (**b**) of cultivation. (1) pH 9 and 0.2 g L^−1^ NaCl, (2) pH 9 and 5 g L^−1^ NaCl, (3) pH 9 and 10 g L^−1^ NaCl, (4) pH 10 and 0.2 g L^−1^ NaCl, (5) pH 10 and 5 g L^−1^ NaCl, (6) pH 10 and 10 g L^−1^ NaCl, (7) pH 11 and 0.2 g L^−1^ NaCl, (8) pH 11 and 5 g L^−1^ NaCl, (9) pH 11 and 10 g L^−1^ NaCl.

**Figure 3:**
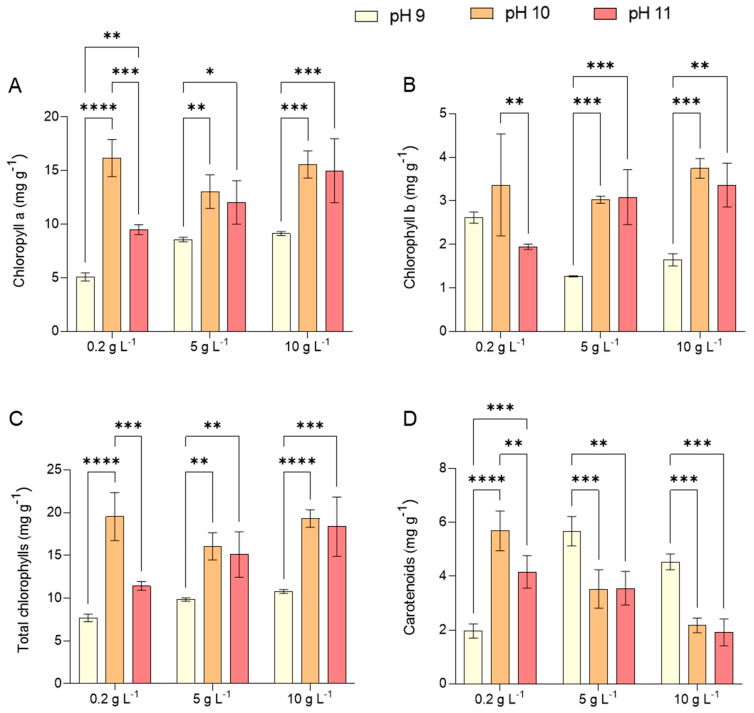
Concentration of chlorophyll a (**A**), chlorophyll b (**B**), total chlorophylls (**C**), and carotenoids (**D**) obtained by *L. platensis* as a function of salt concentration in the media (0.2, 5, and 10 g L^−1^) under three different pH (9, 10, and 11). Mean differences were compared using Tukey’s test (*n* = 3, * *p* < 0.05, ** *p* < 0.01, *** *p* < 0.001, **** *p* < 0.0001).

A moderate growth rate was observed at pH 9, as corroborated by microscopic images of *Limnospira* cells in Figure 2b, which reveal elongated single filaments compared to those observed on day 0 (Figure 2a). These findings align with the study by Babu et al. [26], which explored the influence of alkalinity on *L. platensis*. Although chlorophyll b is not synthesized by cyanobacteria, values were reported here based on absorbance at 649 nm using Arnon’s equations, which are classically applied in studies involving green microalgae. Future studies will employ pigment separation techniques (e.g., HPLC) to validate the specificity of chlorophyll measurements. This was in contrast with the chlorophyll content shown in Figure 3A–C which further supports the stability provided under the condition of pH 10, highlighting their potential suitability for large-scale cultivation of *L. platensis*. 

In this study, *L. platensis* exhibited optimal growth at pH 10, whereas pH 9 was identified as the most favorable for proliferation, as it closely matches the initial pH of the JM medium used in all experiments. These findings reinforce the classification of *L. platensis* as an alkalophilic microorganism, as confirmed by the elongation and proliferation of cell filaments across the pH 9–11 range (Figure 2a,b).

The suitability of pH 10 as the optimal condition for *Limnospira* cultivation is further validated by the highest recorded levels of chlorophyll a and b, total chlorophylls, and carotenoids (Figure 3A–D), across all tested salinity conditions. Similar trends were reported by Carvalho et al. [27] and Kim et al. [28], who identified pH 10 and 10.5, respectively, as the optimal range for maximizing chlorophyll a and protein synthesis. Conversely, Ismaiel et al. [25] recorded peak chlorophyll a and carotenoid concentrations at pH 8.5, measuring 10.6 mg g^−1^ DW and 2.4 mg g^−1^ DW, respectively. It should be noted that the cyanobacterium *Limnospira* does not naturally contain chlorophyll b. Therefore, the signal detected at 649 nm is likely due to experimental overlap or background pigments.

The increase in chlorophyll a content at higher pH levels (>10) is primarily attributed to reduced free CO_2_ availability in the medium, as carbonate becomes the dominant form while bicarbonate serves as the primary carbon source for *L. platensis*. This phenomenon was similarly noted by Sharma et al. [29]. These results emphasize the crucial role of carbon availability in growth regulation, as variations in carbon content significantly impact photorespiration, which is a mechanism that protects the photosynthetic membrane from light-induced damage under conditions of limited carbon assimilation. Notably, *L. platensis* and other cyanobacteria exhibit enhanced photosynthetic efficiency when grown in bicarbonate-enriched media, such as the JM medium used in this study. The rate of CO_2_ fixation in cyanobacteria is directly influenced by their ability to accumulate inorganic carbon sources [30].

Furthermore, *Limnospira* thrives in relatively high pH conditions (9.5–9.8), which provide a natural defense against contamination by competing algae. Maintaining high alkalinity is essential for its growth, with sodium bicarbonate playing a key role in stabilizing the pH. Additionally, increased salinity levels have been shown to enhance electron transport chain activity and photosystem II (PS-II) efficiency by influencing the PS-II reaction center and inducing modifications in the water oxidation complex [31].

Besides its impact on chlorophyll and protein levels, salinity exhibited an inverse correlation with carotenoid content (Figure 3D), indicating a distinct physiological adaptation to saline stress. Specifically, chlorophyll synthesis was suppressed while carotenoid accumulation increased, suggesting a protective response to environmental stressors. This finding is consistent with previous reports demonstrating that the elevated salinity levels reduce chlorophyll content in microalgae [32] and total carotenoids [33]. The optimal productivity for both chlorophylls and total carotenoids is generally observed at low salinity levels (2–3 g L^−1^) in most microalgae. For instance, *Dunaliella salina* exhibited the maximum β-carotene production with 3 g L^−1^ NaCl [33], while *Dunaliella viridis* showed the highest chlorophyll a and total carotenoid concentrations at 2 g L^−1^ NaCl [34].

**Figure 4:**
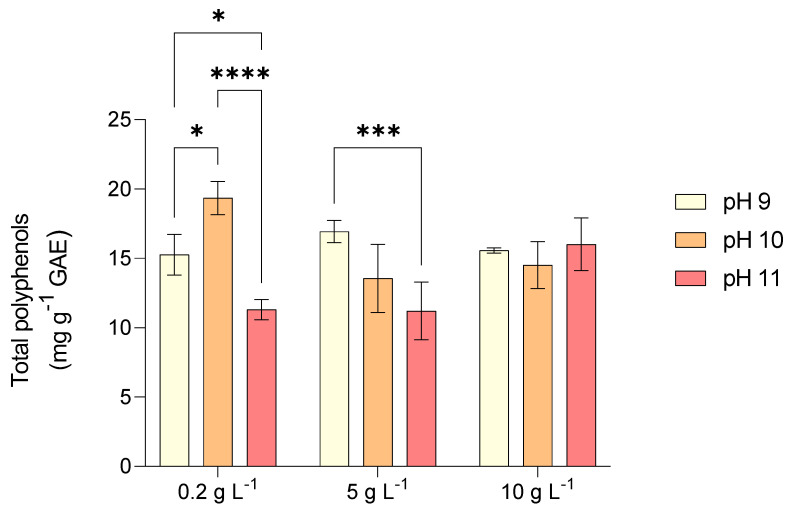
Total polyphenols (TP) content obtained by *L. platensis* as a function of salt concentration in the media (0.2, 5, and 10 g L^−1^) under three different pH levels (9, 10, and 11). Mean differences were compared using Tukey’s test (*n* = 3, * *p* < 0.05, ** *p* < 0.01, *** *p* < 0.001, **** *p* < 0.0001).

**Figure 5:**
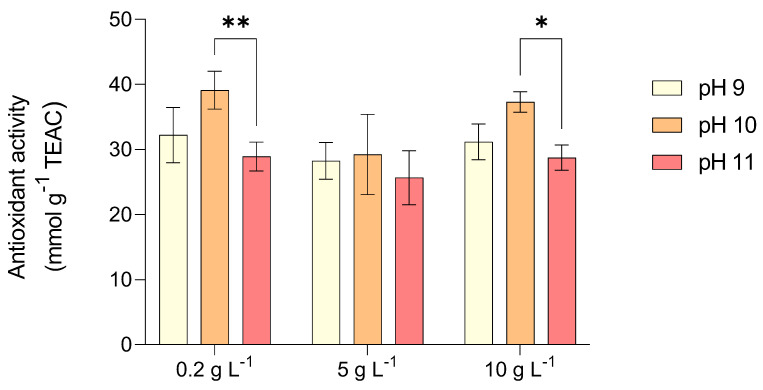
Antioxidant activity obtained by *L. platensis* as a function of salt concentration in the media (0.2, 5, and 10 g L^−1^) under three different pH levels (9, 10, and 11). Mean differences were compared using Tukey’s test (*n* = 3, * *p* < 0.05, ** *p* < 0.01, *** *p* < 0.001, **** *p* < 0.0001).

The highest total polyphenol content in *L. platensis* was recorded in test 2 (pH 10 and 0.2 g L^−1^ NaCl) with 19.5 mg gallic acid equivalents (GAE) gDW^−1^ and in test 4 (pH 9 and 0.2 g L^−1^ NaCl) with 17 mg GAE gDW^−1^, whereas the lowest content (11.5 mg GAE gDW^−1^) was observed in test 3 (pH 11 and with 0.2 g L^−1^ NaCl) and test 6 (pH 11 and with 5 g L^−1^ NaCl) (Figure 4). *L. platensis* exhibited strong antioxidant activity at pH 10 across all tested salinity levels (0.2–10 g L^−1^). However, a significant decline was noted at pH 11, particularly at salinities of 0.2 and 5 g L^−1^ (Figure 5). This reduction in antioxidant activity at higher pH levels may be attributed to the decline of non-enzymatic antioxidants such as phycocyanin and phenolic compounds, as suggested by previous studies [35].

In the present study, increasing pH led to a decline in phycocyanin content, particularly when shifting from pH 10 to 11 under increasing salinity. Likewise, total polyphenol content significantly decreased when pH shifted from 10 to 11 (Figure 4). The reduction in polyphenol production at elevated pH levels aligns with evidence indicating that algal cells begin to degrade under extreme alkaline conditions, potentially leading to the disruption of various cellular functions, including antioxidant defenses [36]. To mitigate oxidative stress, algae produce a range of antioxidant enzymes that play a crucial role in maintaining cellular redox balance. The enhanced antioxidant activity observed at pH 10 might suggest a cooperative function of these enzymes in counteracting oxidative stress under alkaline conditions. Among them, enzymes responsible for neutralizing reactive oxygen species (ROS) help detoxify harmful radicals and prevent oxidative damage [37]. Some enzymes play a primary role in converting superoxide radicals into less harmful molecules, while others regulate hydrogen peroxide levels by catalyzing its breakdown into water and oxygen. A decline in the activity of specific antioxidant enzymes at elevated pH may indicate their sensitivity to extreme alkaline stress. The relatively stable activity of certain antioxidant enzymes between pH 8.5 and 10 suggests that cells may rely on specific enzymes for hydrogen peroxide detoxification rather than alternative pathways. Additionally, increased antioxidant enzyme activity at a more neutral pH (7.5) may help counteract ROS generated through photosynthesis [38].

### 2.3. Proximate Biomass Composition of L. platensis Under Salinity and Alkalinity

**Figure 6:**
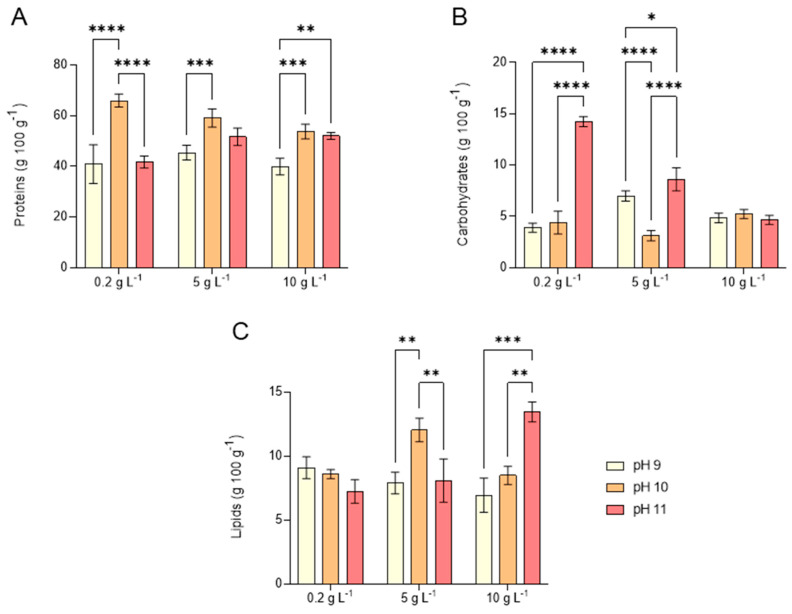
Total protein (**A**), total carbohydrates (**B**), and total lipids (**C**) content obtained by *L. platensis* as a function of salt concentration in the media (0.2, 5, and 10 g L^−1^) under three different pH levels (9, 10, and 11). Mean differences were compared using Tukey’s test (*n* = 3, * *p* < 0.05, ** *p* < 0.01, *** *p* < 0.001, **** *p* < 0.0001).

The effect of alkalinity and salinity on the biomass composition of *L. platensis* in terms of macronutrients, such as total proteins (TP), total carbohydrates (TC), and total lipids (TL), is summarized in Figure 6.

As a rule of thumb, TP constituted the largest fraction (approximately 50%), followed by TL and TC under all tested conditions, except for test 7, where TC was significantly higher than under other salinity and alkalinity conditions. This macronutrient distribution aligns with findings for other microalgae species, such as *Tetraselmis chuii*, *Chlorella vulgaris*, and *Isochrysis galbana* [39].

The TP fraction ranged between 40% and 60% across all salinity and alkalinity conditions (Figure 6A). A significant increase in TP was observed at pH 10 under the lowest salinity conditions tested. However, the shift in salinity from 5 to 10 g L^−1^ did not have a significant influence on TP content either at pH 10 or 11. Regarding TC (Figure 6B), cultivation at pH 11 resulted in a significant increase in TC content at 0.2 g L^−1^ NaCl compared to pH 9 and 10 (tests 1 and 4, respectively), with TC reaching 14%, substantially higher than the average TC content of 5% recorded under other conditions. A similar but less pronounced trend was observed at 5 g L^−1^ NaCl (tests 2, 5, and 8). However, at 10 g L^−1^ NaCl, no significant differences in TC were detected across the pH range tested (tests 3, 6, and 9).

The TL fraction ranged between 7% and 13% across the investigated salinity and alkalinity conditions (Figure 6C). While low salinity levels had no significant impact on TL content, tests 5 (5 g L^−1^ NaCl at pH 10) and 9 (10 g L^−1^ NaCl at pH 11) showed a significant increase in TL fraction. This effect was particularly evident in test 9, where TL content doubled compared to test 3, highlighting the influence of increasing pH from 9 to 11.

This macronutrient distribution, characterized by a remarkable protein fraction, differs significantly from the commonly reported chemical composition of *L. platensis*, which typically consists of 15–25% carbohydrates, 55–70% proteins, and 4–7% lipids [40]. The balance between lipid and carbohydrate synthesis is influenced by shared biosynthetic pathways, which can be redirected towards either component under specific conditions [41]. This may explain the low carbohydrate content observed in the present study compared to the lipid across the tested pH range. Research indicates that microalgae grown under moderately saline conditions accumulate carbohydrates as an adaptation strategy to fluctuations in salinity [42]. This could account for the elevated carbohydrate levels at low salinity (0.2 g L^−1^) compared to higher salinities when *L. platensis* was grown at pH 11 (Figure 6B). Nonetheless, the carbohydrate levels differ from one species to another [43].

The impact of salinity on the biochemical composition of biomass remains an area of limited understanding. Some studies indicate a direct correlation between increased salinity and enhanced lipid accumulation in *L. platensis* [44]. These findings seem in part corroborated by this study, particularly in terms of TL modifications at the highest pH levels (Figure 6C). Salinity is known to influence trichome structure and alter the biochemical profile of biomass, suggesting a potential approach to tailor these characteristics. These adaptations become more evident once the cells have adapted to higher salinity levels. However, further research is needed to clarify how salt stress, beyond initial acclimation, affects both the biochemical composition and growth rate in *L. platensis* [16]. In microalgae cultivation, adjusting factors such as salinity, pH, nutrient levels, and the operational trophic mode represent an effective bioengineering strategy to boost both biomass yield and composition [45]. While not all microalgal strains tolerate high salinity, *L. platensis* is known to prosper at typical seawater salinity (around 35 g L^−1^), and the strain used in this study demonstrated tolerance up to 41 g L^−1^ [12]. Moreover, previous research suggests that biomass production remains stable at salinities up to 40 g L^−1^ NaCl, though a slight decline occurs at 60 g L^−1^ NaCl [16].

### 2.4. Phycocyanin Production by L. platensis Under Alkalinity and Salinity

**Figure 7:**
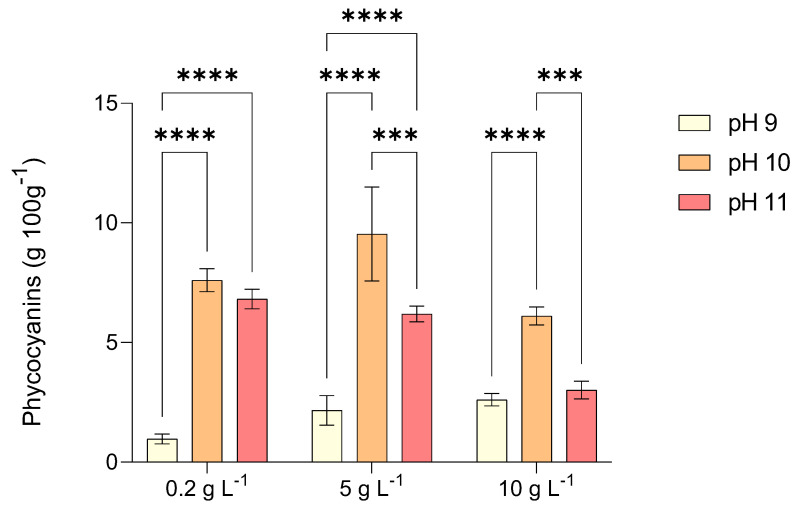
Concentration of phycocyanin (PC) obtained by *L. platensis* as a function of salt concentration in the media (0.2, 5, and 10 g L^−1^) under three different pH levels (9, 10, and 11). Mean differences were compared using Tukey’s test (*n* = 3, * *p* < 0.05, ** *p* < 0.01, *** *p* < 0.001, **** *p* < 0.0001).

Figure 7 depicts the concentration of phycocyanin (PC) in extracts from *L. platensis* biomass cultivated under photoautotrophic conditions with increasing levels of salinity and alkalinity.

Interestingly, PC production showed an even more pronounced dependence on environmental conditions. Maximum PC content (9.54 g 100 g^−1^ dry weight) was achieved at pH 10 and 5 g L^−1^ NaCl (Test 5), more than tripling the concentration obtained at pH 9 under comparable salinity. Moderate salinity appears to act as a mild stressor that triggers the upregulation of phycobiliprotein biosynthesis, potentially as a photoprotective and antioxidant response. At higher salinity (10 g L^−1^ NaCl) and extreme alkalinity (pH 11), PC levels declined, likely due to pigment degradation and reduced energy allocation to non-essential pathways under severe stress.

Research indicates that the rise in PC production results from a dynamic interplay among culture medium composition, the availability of a carbon source, and the adaptive physiological reactions of microalgae to tailored culture conditions. The physiological mechanism behind the trend observed in this study can be attributed to the salt-induced osmotic adjustment combined with alkalinity-driven modulation of carbon metabolism. Moderate osmotic stress can enhance pigment production as part of the cellular antioxidant system, whereas excessive stress impairs the electron transport chain, leading to oxidative damage and reduced pigment biosynthesis. These factors collectively induce a stress-driven environment that enhances PC synthesis [46].

In this study, it was evident that pH in the range 10–11 had a significant effect in increasing PC concentration compared to pH 9. Specifically, test 5 showed the highest total PC content at 9.54 g 100 g^−1^, while the lowest PC content of 0.97 g 100 g^−1^ was expressed by test 1. These findings are consistent with the trend observed by other researchers. Deshmukh and Puranik [47] studied the PC production by *Synechocystis* sp. within a wide range of pH (5–12). They achieved a maximum PC production of 14.5 mg L^−1^ at pH 10 using a BG-11 medium. Poza-Carrión et al. [48] investigated how pH, irradiance, and inorganic carbon availability collectively influence the growth and pigment composition of the cyanobacterium *Nostoc* sp. strain UAM206. Their findings revealed a direct correlation between rising pH levels and increased concentrations of PC, phycoerythrin, and allophycocyanin.

Regarding the correlation between salinity and PC production, the addition of salt resulted in a slight, gradual increase in PC for the lowest pH (tests 1, 4, 7) and in a more consistent increase for pH 10 (tests 2 and 5). These findings agree with Abd El-Baky [49], who observed that elevating salinity levels in the nutrient medium resulted in a substantial enhancement of phycocyanin accumulation and the overall soluble protein content in *Spirulina maxima*. On the other hand, at the highest level of pH, a decrease of PC content was observed, which was more evident in test 9. This trend aligns with observed behavior in *L. platensis*, where the addition of varying NaCl concentrations led to higher protein and PC content [14,44].

Salinity plays a crucial role in regulating pigment biosynthesis in microalgae. It has been hypothesized that phycobilisome detachment from the thylakoid membrane may occur because of energy transfer from phycobiliproteins to the photosynthetic reaction center and the assimilation of other nutrients found in the growth medium [50,51]. Given that microalgae thrive in saline environments, osmosis significantly influences pigment content. Elevated NaCl concentrations create a hypertonic extracellular environment, inducing a net efflux of water molecules from the cell, leading to cellular shrinkage and structural damage [52]. Some studies have demonstrated that NaCl concentration in the range 10–15 g L^−1^ enhances phycobiliprotein synthesis in cyanobacteria. Hemlata and Fatma [53] reported for *Anabaena* NCCU-9 the best phycobiliprotein yield of 135.73 mg g^−1^ at 10 g L^−1^ of NaCl, while for the cyanobacterium *Oscillatoria* sp., it has been reported an increased content of phycoerythrin up to 66.7 mg g^−1^ with 15 g L^−1^ NaCl [54].

The applicability of PC across different domains is primarily determined by its purity, which is conventionally evaluated through an absorbance ratio. This ratio compares the absorbance of PC at 620 nm (A620) to that of other proteins at 280 nm (A280). The extraction purity (EP) metric, defined as the A620/A280 ratio, categorizes PC based on its suitability for various applications. A PC sample with an EP of 0.7 or greater is designated as food grade, rendering it appropriate for use as a food additive or a natural blue pigment in cosmetic formulations. When the EP ranges from 0.7 to 3.9, PC is classified as reagent grade, with values of 1.5 or higher being particularly suitable for cosmetic applications. An EP exceeding 4 indicates analytical-grade purity, making the PC suitable for pharmaceutical applications [55].

**Figure 8:**
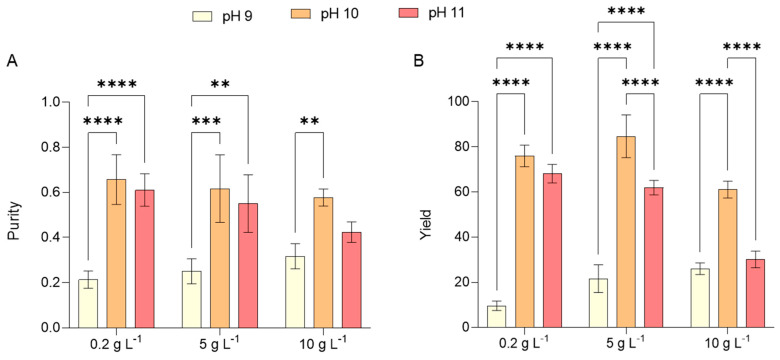
Purity (**A**) and yield (**B**) of phycocyanin (PC) obtained by *L. platensis* as a function of salt concentration in the media (0.2, 5, and 10 g L^−1^) under three different pH levels (9, 10, and 11). Mean differences were compared using Tukey’s test (*n* = 3, * *p* < 0.05, ** *p* < 0.01, *** *p* < 0.001, **** *p* < 0.0001).

In the current study, PC purity ranged between 0.21 with test 1 and 0.66 with test 2. Across all the salinity levels investigated, lower levels of purity were obtained with pH 9, while better results were achieved with pH 10 (Figure 8A). Correspondingly, the PC yields reflected the same trend with respect to variations in salinity and alkalinity, with the highest yield of 84.73 mg g^−1^ and the lowest of 9.65 mg g^−1^ obtained with tests 5 and 1, respectively (Figure 8B). These results are consistent with those documented by Russo et al. [12], who cultivated *L. platensis* using an organic substrate (brewery wastewater) combined with seawater at a 2% *v v^-1^* ratio within the JM control medium, identical to that employed in this study. Their findings indicated a purity level of 0.7 and a PC yield of 35 mg g^−1^ when cultivated with 1 g L^−1^ of NaCl, that is the concentration of salt in the JM. These values were three and four times greater, respectively, than those recorded in this study under test 1, where the NaCl concentration was maintained at 0.2 g L^−1^, five times lower than that of the JM medium.

The purity of PC is strongly dependent on the extraction techniques employed, which are regulated by various physical and chemical factors, including temperature, pH, solvent selection, biomass-to-solvent ratio, and the physical state of the biomass (fresh or dried). Enhancing PC yield can be achieved through two primary approaches: optimizing the extraction process or exposing *Limnospira* sp. to abiotic stress conditions. Notably, a study subjecting *Limnospira* biomass to a freeze-thaw treatment in conjunction with a pulsed electric field led to a significant increase in PC yield, reaching 147.33 mg g^−1^ [56].

The market value of PC is highly dependent on its purity, with prices escalating as purity levels increase, particularly in sectors such as cosmetics, agro-chemistry, and food industries [57]. Literature reports indicate that analytical-grade PC, characterized by a purity level above 4, can be priced at approximately 60 USD per gram, while the highest purity variants may reach an astonishing 19,500 USD per gram. Recent research also suggests that PC utilized as a biocolorant is available at around 0.35 USD per gram, whereas analytical-grade PC can command prices of up to 4500 USD per gram [58]. Projections estimate that the global PC market will expand to 245.5 million USD by 2027 and further to 279.6 million USD by 2030 [59]. These elevated costs stem from the complexities associated with the extraction and purification of PC, positioning it as a highly valuable yet costly protein pigment.

The findings of this study highlight that finely tuning environmental parameters, rather than applying extreme stress, is crucial in optimizing both biomass yield and biochemical quality while promoting sustainable and eco-friendly production practices.

## 3. Materials and Methods

### 3.1. Inocula and Culture Media Preparation

The strain *Limnospira platensis* SAG 21.99 used in this study was sourced not axenically from the culture collection of algae at the Gottingen University, Germany. The cell cultures were maintained and grown in a modified version of the Jourdan Medium (JM), composed as follows in g L^−1^: 5 NaHCO_3_; 1.6 KOH, 5 NaNO_3_; 0.027 CaCl_2_·2H_2_O; 0.4 K_2_SO_4_, 2 K_2_HPO_4_; 1 NaCl; 0.4 MgSO_4_·7H_2_O; 0.16 EDTA-Na_2_; 0.01 FeSO_4_·7H_2_O; and 1 mL of Trace elements. The Trace elements solution was prepared with the following composition (mg L^−1^): 250 EDTA-Na_2_; 57 H_3_BO_3_; 110 ZnSO_4_·7H_2_O; 25.3 MnCl_2_·4H_2_O; 8.05 CoCl_2_·6H_2_O; 7.85 CuSO_4_·5H_2_O; 5.5 Mo_7_O_24_ (NH_4_)_6_·4H_2_O. The initial natural pH of the JM medium was approximately 9.0, and its baseline salinity was ~1 g L^−1^ NaCl. Erlenmeyer flasks of 150 mL were filled with 50 mL of JM medium, inoculated with approximately 10 mL of microalgae, sealed with a cotton plug, and continuously illuminated at room temperature by white, fluorescent lamps (Model T8 36 W IP20, CMI, Bergisches Land, Germany) providing a light intensity of 50 µmol m^−2^ s^−1^, measured with a luxmeter (Model HD 2302.0, Delta OHM, Padua, Italy). The inoculum was cultivated for about one week, until the end of the exponential growth phase, before being used for experiments.

### 3.2. Cultivation Conditions and Experimental Setup

*L. platensis* was cultivated under strictly photoautotrophic batch conditions in 20 L transparent cylindrical PVC photobioreactors (hereafter referred to as PBRs), which allowed uniform light penetration throughout the culture volume. Each PBR had an outer diameter of 16 cm, an inner diameter of 14 cm, and a height of 27 cm. Illumination was provided externally using four warm white LED panels (model 2835, 30 W IP33, ImportLed, Shenzhen, China), providing a light intensity of 150 µmol m^−2^ s^−1^. This external setup ensured homogeneous light distribution without creating localized overheating or photoinhibition zones. Filtered compressed air, provided by an air pump (GIS Air Compressor, Carpi, MO, Italy), was continuously supplied to the PBRs through a perforated rubber, to ensure homogenous mixing and prevent sedimentation. A 12 h light/12 h dark photoperiod was adopted to simulate natural diel cycles, which are commonly encountered in large-scale open or semi-closed systems. This photoperiod was chosen to promote physiological recovery during the dark phase, reduce photoinhibition risk under constant illumination, and better replicate real-world operational conditions for scalable outdoor or hybrid PBRs. Such cycling is also known to regulate circadian rhythms and influence metabolite production in cyanobacteria, making it a more ecologically relevant and industrially translatable cultivation strategy. PBRs were filled with 3 L of working volume and inoculated at an initial biomass concentration of 0.3 g L^−1^.

To investigate the effects of salinity and alkalinity (simulated by pH variation) on *L. platensis* growth and biochemical composition, three pH levels (9, 10, and 11) and three NaCl concentrations (0.2, 5, and 10 g L^−1^) were tested in combination, as shown in Table 2.

These pH values were selected to explore a realistic alkaline range suitable for cyanobacterial growth, while NaCl concentrations represented sub-natural (0.2 g L^−1^), moderately elevated (5 g L^−1^), and highly elevated (10 g L^−1^) salinity levels compared to the standard JM medium (1 g L^−1^). The lowest salinity treatment (0.2 g L^−1^ NaCl) was selected to simulate a low-stress condition below the standard 1 g L^−1^ L NaCl of the JM medium. While not a true control, this allowed assessing salinity-driven physiological shifts. Future studies will include a baseline control at 1 g L^−1^ NaCl for direct comparison. The choice of these specific values allowed the evaluation of moderate stress effects without inducing lethal shock, thereby simulating industrially relevant cultivation stresses. The PBRs operated in batch mode, the tests were carried out in triplicate, and lasted 5 days. The pH was initially adjusted with KOH or HCl prior to inoculation. It should be noted that despite careful initial pH setting, a moderate drift toward pH 10 was observed during the 5-day cultivation, mainly due to CO_2_ uptake associated with photosynthetic activity. Active pH control systems were not employed to maintain the natural response of the culture to environmental changes, although future experiments could benefit from continuous pH regulation. Microalgae growth was monitored by measuring optical density and biomass concentration. After cultivation, the final dry weight (g L^−1^) was determined, and a biomass analysis was performed.

### 3.3. Cell Growth and Dry

*L. platensis* growth was evaluated by monitoring the optical density (OD) of the culture at 560 nm using a spectrophotometer (model ONDA V30 SCAN–UV VIS, ZetaLab, Padua, Italy). A regression equation was calculated to describe the relationship between dried biomass concentration and OD. Dry biomass concentration was evaluated gravimetrically as follows; (a) a known volume (10 mL) of culture (V) was drawn from the PBRs, (b) the sample was filtered through a pre-weighted (W1) glass microfiber filter (GF/CTM 55 mm diameter, Whatman, Incofar Srl., Modena, Italy), and the biomass retained on the filter was dried at 105 °C overnight to a constant weight (W2), (c) the filter paper had been previously dried in a forced-air oven (model 30, Memmert Gmbh, Scwabach, Germany) at 105 °C for 2 h, then cooled to room temperature in a desiccators, and weighed using an analytical scale (model M, Bel Engineering Srl, Monza, MI, Italy). The final biomass concentration (dry weight), $X_{f}$ (g L^−1^), was calculated using the following equation:

(1) $$X_{f}=\frac{{\bar{W}}_{f}}{V_{s}}$$

where, ${\bar{W}}_{f}$ = average (from triplicate sample) experimental weight (g) of dried algal biomass at final time of culture, and $V_{s}$ = volume (L) of sample of algae culture used for the test at the final time.

The difference in biomass concentration ΔX is represented by the following equation:

(2) $$\Delta X=X_{f}-X_{0}$$

where, $X_{f}$ is the final biomass concentration (g L^−1^) obtained at ($t_{f}$) and $X_{0}$ is the initial biomass concentration (g L^−1^) obtained at ($t_{0}$).

The average volumetric biomass productivity ($Q_{X}$) was expressed as follows:

(3) $$Q_{X}=\frac{X_{f}-X_{0}}{t_{f}-t_{0}}=\frac{\Delta X}{\Delta t}$$

where, $X_{f}$ and $X_{0}$ are the same as those variables previously presented.

The average specific growth rate ($\mu_{av}$) was calculated according to the following equation:

(4) $$\mu_{av}=\frac{\ln\left(\frac{X_{f}}{X_{0}}\right)}{t_{f}-t_{0}}=\frac{\ln\left(\frac{X_{f}}{X_{0}}\right)}{\Delta t}$$

where, $X_{f}$, $X_{0}$, $t_{f}$ and $t_{0}$ are the same variables of Equation (3).

The pH of culture suspensions was measured by a pH-meter (model HI 2210, Hanna Instruments, Woonsocket, RI, USA).

### 3.4. Analysis of Proximate Composition

#### 3.4.1. Protein Analysis

Protein quantification was performed according to the method of Lowry et al. [60], with slight modifications. Briefly, 2 mg of freeze-dried *L. platensis* biomass was suspended in 5 mL of distilled water. From this suspension, 0.5 mL was used for protein determination. Prior to the assay, two reagents were prepared: Reagent 1, consisting of 1% (*w*/*v*) potassium sodium tartrate, and Reagent 2, prepared by dissolving 2 g of sodium carbonate in 100 mL of 0.1 N sodium hydroxide. For the reaction mixture, 50 mL of Reagent 2 and 1 mL of Reagent 1 were combined. For the assay, 0.5 mL of sample solution was mixed with 0.5 mL of 1 N sodium hydroxide and incubated in a water bath at 100 °C for 5 min to ensure protein solubilization. After cooling to room temperature, 2.5 mL of the prepared mixture was added and allowed to react for 10 min. Subsequently, 0.5 mL of Folin–Ciocalteu reagent was added, and the mixture was incubated in the dark at room temperature for 30 min to complete color development. Absorbance was measured at 750 nm using a UV–Vis spectrophotometer (Multiskan™ SkyHigh, Thermo Fisher Scientific Inc., Milan, Italy). A calibration curve was generated using a bovine serum albumin (BSA) stock solution. Standard solutions were processed in parallel with the samples, following the same protocol. Protein concentration in each sample was determined based on the standard curve and expressed as BSA equivalents. All analyses were conducted in triplicate, and results are presented as mean ± standard deviation (SD).

#### 3.4.2. Lipid Analysis

Lipid extraction was carried out following Bligh and Dyer [61] and Folch et al. [62] with minor modifications. Briefly, 2 mg of freeze-dried *L. platensis* biomass was suspended in 1.5 mL of 1 N sodium hydroxide containing 25% methanol and incubated at 100 °C for 30 min. After cooling, 3 mL of methanol:chloroform (1:2, *v*/*v*) and 0.5 mL of 0.9% NaCl were added, and the mixture was vortexed thoroughly. Phase separation was achieved by centrifugation at 4 °C for 10 min at moderate speed. The lower chloroform phase (1 mL) was collected, evaporated under a nitrogen stream, and treated with 100 µL of concentrated sulfuric acid. Following thermal treatment, 2.4 mL of 68% (*w*/*v*) phosphovanillin reagent was added, and the mixture was incubated at room temperature for 10 min. Absorbance was measured at 530 nm using a spectrophotometer (Multiskan™ SkyHigh, Thermo Fisher Scientific Inc., Milan, Italy), and lipid content was quantified using an external calibration curve generated with a 99% fat standard oil. All measurements were performed in triplicate, and results were reported as mean ± standard deviation (SD).

#### 3.4.3. Carbohydrate Analysis

Carbohydrate quantification was performed following the method of Dubois et al. [63], with slight modifications. For each analysis, 2 mg of freeze-dried *L. platensis* biomass was suspended in 5 mL of distilled water. A 5% (*w*/*v*) phenol solution and concentrated sulfuric acid were freshly prepared prior to the assay. Briefly, 0.2 mL of the sample solution was combined with 0.2 mL of 5% phenol solution, followed by the rapid addition of 1 mL of concentrated sulfuric acid. The reaction mixture was cooled in an ice-water bath to stabilize the reaction. Absorbance was measured at 488 nm using a spectrophotometer (Multiskan™ SkyHigh, Thermo Fisher Scientific Inc., Milan, Italy). A calibration curve was made from a glucose standard stock solution. Standard solutions were processed in parallel with the samples under identical conditions. Carbohydrate concentrations in the samples were calculated based on the standard curve and expressed as glucose equivalents. All measurements were performed in triplicate, and results were reported as mean ± standard deviation (SD).

### 3.5. Determination of Photosynthetic Pigment Content

A total of 2 mg of algal dry powder was suspended in 1.5 mL of 70% ethanol, then vortexed and incubated in a boiling water bath for 15 min. The mixture was subsequently centrifuged at 4000 rpm for 5 min. The OD of the supernatant was measured at 665 nm, 649 nm, and 470 nm to quantify chlorophyll a, chlorophyll b, and carotenoids, respectively. The concentrations of these photosynthetic pigments were then calculated using the mathematical equation described by Arnon et al. [64].

(5) $$Chl_{a}\left(\frac{\mathrm{mg}}{L}\right)=\frac{\left(13.95\;A_{665}-6.88\;A_{649}\right)\;V_{2}}{V_{1}}$$

(6) $$Chl_{b}\left(\frac{\mathrm{mg}}{L}\right)=\frac{\left(24.96\;A_{649}-7.32\;A_{665}\right)\;V_{2}}{V_{1}}$$

(7) $$C_{carotenoid}\left(\frac{\mathrm{mg}}{L}\right)=\frac{\left(1000\;A_{470}-2.05\;C_{a}-114.8\;C_{b}\right)\;V_{2}}{245\;V_{1}}$$

where *V*_1_ and *V*_2_ represent the volumes of the sampled *L. platensis* suspension and its subsequent supernatant, respectively, A665, A649, and A470 denote the absorbance values of the supernatant at 665 nm, 649 nm, and 470 nm, respectively. *Chl_a_*, *Chl*_b_, and carotenoid stand for the contents of chlorophyll a, chlorophyll b, and carotenoids, respectively. It should be noted that *L. platensis*, being a cyanobacterium, does not naturally produce chlorophyll b. Therefore, any absorbance detected at 649 nm and attributed to chlorophyll b may result from spectral interference or pigment degradation products. The values reported should be interpreted with caution and are retained here primarily for comparative purposes using standard methods

### 3.6. Antioxidant Power Determination

The DPPH (2,2-Diphenyl-1-picrylhydrazyl) assay was performed following a slightly modified protocol based on Brand-Williams et al. [65]. Approximately 2 mg of lyophilized algae powder was dispersed in 1 mL of ethanol and sonicated for 1 min at 80% power using a Bandelin Sonoplus HD 4100 B and Co. KG, Berlin, Germany). The mixture was then centrifuged at 4000 rpm for 5 min at 20 °C. Subsequently, 50 μL of the extract or the standard (Trolox) was added to 2 mL of a methanolic solution of DPPH at a concentration of 40 μM. After a 60-minute incubation period at room temperature, the OD was measured against a blank at 517 nm using 1 cm wide disposable cuvettes in a spectrophotometer Multiskan™ SkyHigh (Thermo Fisher Scientific Inc., Milan, Italy). Quantitative analysis was carried out using an external standard calibration method, and the results were expressed in mM g^−1^ of TEAC (Trolox equivalent antioxidant capacity). All measurements were performed in triplicate, and results were reported as mean ± standard deviation (SD).

### 3.7. Total Polyphenols Determination

The total polyphenol content was determined using the Folin–Ciocalteu method, following a modified protocol based on Singleton [66]. For each measurement, 100 μL of the ethanolic extract (70:30) (2 mg of biomass in 1 mL) or the standard (gallic acid) was mixed with 500 μL of Folin–Ciocalteu reagent and incubated at room temperature for 5 min. Subsequently, 3 mL of 10% *w*/*v* Na_2_CO_3_ and ultrapure water were added to achieve a final volume of 10 mL. After 90 min of incubation at room temperature, the OD was measured at 725 nm against a blank using a Multiskan™ SkyHigh spectrophotometer (Thermo Fisher Scientific Inc., Milan, Italy) and 1 cm wide disposable cuvettes. Quantitative analysis was performed using an external standard calibration method, with the results expressed as mg g^−1^ of gallic acid equivalent (GAE). All measurements were performed in triplicate, and results were reported as mean ± standard deviation (SD).

### 3.8. Statistical Analysis

Data on *L. platensis* growth, pigments, and proximate analysis composition were analyzed using a two-way ANOVA with Tukey’s post hoc test, employing GraphPad PRISM 9.00 (GraphPad Software, San Diego, CA, USA). The analysis considered salinity (0.2, 5, and 10 g L^−1^) and pH (9, 10, and 11) as independent factors to assess their effects on the measurements. Multiple comparisons were conducted using Tukey’s test with a 95% confidence interval. Significance levels, indicated by asterisks (*), are based on *p*-values. No asterisks denote a *p*-value > 0.05; * indicates *p*-value < 0.05; ** indicates *p*-value < 0.01; *** indicates *p*-value < 0.001; and **** indicates *p*-value < 0.0001. Each experimental condition was investigated in triplicate.

## 4. Conclusions

This study demonstrates that moderate modulation of salinity and pH can be effectively used to optimize biomass productivity, biochemical composition, and phycocyanin content in *Limnospira platensis* cultivated under photoautotrophic batch conditions. The highest biomass yield (1.596 g L^−1^) was recorded at pH 11 with 10 g L^−1^ NaCl, while the optimal phycocyanin concentration (9.54 g 100 g^−1^) occurred at pH 10 and 5 g L^−1^ NaCl.

Biochemical composition analysis showed a predominant protein fraction (40–60%) across all conditions, reinforcing *L. platensis*’s value as a protein-rich biomass. Meanwhile, total lipid content increased significantly under extreme alkalinity at pH 11, particularly in combination with 10 g L^−1^ NaCl, which may have implications for biofuel production. Polyphenol content, known for its antioxidant properties, reached its highest level (19.5 mg GAE gDW^−1^) at pH 10 with low salinity but declined at pH 11, likely due to cellular stress responses. Similarly, antioxidant activity was strongest at pH 10 across all salinity levels but decreased at pH 11, suggesting that while alkaline conditions stimulate stress responses beneficial for bioactive compound synthesis, excessive pH shifts may hinder their accumulation. This study highlights the importance of *L. platensis* as a natural source of powerful antioxidants, especially in varying pH environments. Its ability to boost antioxidant production under stress conditions suggests promising applications in pharmaceuticals, food, cosmetics, and other industries.

These findings emphasize that carefully calibrated environmental stress, rather than extreme conditions, can enhance both biomass quality and industrial utility. Mechanistically, the observed responses can be linked to osmotic adjustment processes, modulation of antioxidant defenses, and the stabilization of photosynthetic activity under stress. However, deeper molecular insights into stress adaptation mechanisms remain unexplored.

Future research should integrate omics technologies, including transcriptomics, proteomics, and metabolomics, to unravel the regulatory networks activated under salinity and alkalinity stress. Additionally, incorporating real-time pH control systems could allow regulating in a better way culture conditions, minimizing pH drift, and enabling a more accurate assessment of pH-specific effects.

Finally, scaling up these findings to industrial PBR systems will require economic analyses of salinity management strategies, carbon supplementation methods, and downstream processing of high-value products like PC. These efforts will be crucial for translating laboratory-scale optimizations into commercially viable, sustainable microalgal production processes in food, pharmaceuticals, and sustainable bioproducts markets. 

## Figures and Tables

**Table 1:** Effect of pH and salinity on growth kinetics parameters.

	pH_final_ /	X_f_ g L^−1^	Q_x_ mg L^−1^ day^−1^	ΔX g L^−1^	µ_av_ day^−1^
Test 1	10.43 ± 0.10	1.299 ± 0.277	135 ± 0.024	0.676 ± 0.120	0.152 ± 0.035
Test 2	10.32 ± 0.12	1.058 ± 0.196	123 ± 0.040	0.613 ± 0.200	0.172 ± 0.045
Test 3	10.20 ± 0.20	1.255 ± 0.216	107 ± 0.029	0.534 ± 0.144	0.123 ± 0.057
Test 4	10.34 ± 0.08	1.225 ± 0.246	83 ± 0.037	0.417 ± 0.184	0.081 ± 0.024
Test 5	10.20 ± 0.10	1.217 ± 0.104	128 ± 0.026	0.642 ± 0.128	0.082 ± 0.014 *
Test 6	10.24 ± 0.08	1.392 ± 0.101	144 ± 0.041	0.721 ± 0.205	0.109 ± 0.012
Test 7	10.63 ± 0.15	1.215 ± 0.175	87 ± 0.064	0.435 ± 0.019	0.090 ± 0.069
Test 8	10.47 ± 0.25	1.142 ± 0.188	82 ± 0.036	0.408 ± 0.181	0.077 ± 0.009 *
Test 9	10.38 ± 0.14	1.596 ± 0.175	94 ± 0.002	0.470 ± 0.012	0.145 ± 0.062

Note: X_f_ = final biomass concentration, Q_x_ = volumetric biomass productivity, ΔX = difference in average biomass concentration, µ_av_ = average specific growth rate, * indicates *p*-value < 0.05.

**Table 2:** Experimental setup showing the combination of pH and NaCl investigated.

Test Number	Initial pH	NaCl Concentration (g L^−1^)	Condition Description
Test 1	9	0.2	Low salinity, baseline alkalinity
Test 2	9	5	Moderate salinity, baseline alkalinity
Test 3	9	10	High salinity, baseline alkalinity
Test 4	10	0.2	Low salinity, baseline alkalinity
Test 5	10	5	Moderate salinity, baseline alkalinity
Test 6	10	10	High salinity, baseline alkalinity
Test 7	11	0.2	Low salinity, baseline alkalinity
Test 8	11	5	Moderate salinity, baseline alkalinity
Test 9	11	10	High salinity, baseline alkalinity

## Data Availability

Data will be available upon request.
